# A Modified Two-Way Active Avoidance Test for Combined Contextual and Auditory Instrumental Conditioning

**DOI:** 10.3389/fnbeh.2021.682927

**Published:** 2021-06-21

**Authors:** Houssein Salah, Ronza Abdel Rassoul, Yasser Medlej, Rita Asdikian, Helene Hajjar, Sarah Dagher, Mouhamad Darwich, Christina Fakih, Makram Obeid

**Affiliations:** ^1^Department of Anatomy, Cell Biology and Physiological Sciences, American University of Beirut, Beirut, Lebanon; ^2^Neuroscience Research Center, Faculty of Medical Sciences, Lebanese University, Hadath, Lebanon; ^3^Division of Child Neurology, Department of Pediatrics and Adolescent Medicine, American University of Beirut Medical Center, Beirut, Lebanon

**Keywords:** two-way active avoidance, contextual conditioning, auditory conditioning, behavior, amygdala, hippocampus

## Abstract

Available two-way active avoidance paradigms do not provide contextual testing, likely due to challenges in performing repetitive trials of context exposure. To incorporate contextual conditioning in the two-way shuttle box, we contextually modified one of the chambers of a standard two-chamber rat shuttle box with visual cues consisting of objects and black and white stripe patterns. During the 5 training days, electrical foot shocks were delivered every 10 s in the contextually modified chamber but were signaled by a tone in the plain chamber. Shuttling between chambers prevented an incoming foot shock (avoidance) or aborted an ongoing one (escape). During contextual retention testing, rats were allowed to freely roam in the box. During auditory retention testing, visual cues were removed, and tone-signaled shocks were delivered in both chambers. Avoidance gradually replaced escape or freezing behaviors reaching 80% on the last training day in both chambers. Rats spent twice more time in the plain chamber during contextual retention testing and had 90% avoidance rates during auditory retention testing. Our modified test successfully assesses both auditory and contextual two-way active avoidance. By efficiently expanding its array of outcomes, our novel test will complement standard two-way active avoidance in mechanistic studies and will improve its applications in translational research.

## Introduction

Learning to recognize and to adaptively avoid threatening, potentially harmful scenarios are two complementary learning mechanisms that are essential for survival. Animal research that investigates disturbances in the neuronal circuitries underlying fear conditioning and avoidance shed light on human conditions reminiscent of anxiety disorders and maladaptive avoidance such as phobias and post-traumatic stress-disorder (PTSD) (LeDoux et al., 2017). In Pavlovian fear or threat conditioning, a neutral conditioned stimulus (CS), such as a tone, light, or a specific context, is paired with an aversive event, most commonly a painful electrical foot shock: the unconditioned stimulus (US). In these testing paradigms, animals learn to recognize a threat or a danger-predicting scenario (the CS) by associating it with an upcoming aversive US, leading to reactive freezing upon reexposure to the CS (2). While classical Pavlovian conditioning paradigms assess the emergence of CS-induced reactive innate defensive freezing, more elaborate adaptive behavioral responses are acquired in the instrumental types of conditioning such as the two-way active avoidance (TAA) test. In standard TAA testing, animals learn to prevent (avoidance) tone-signaled electrical foot shocks by shuttling via a door that separates two adjacent compartments (LeDoux et al., 2017; Medlej et al., 2019a; Salah et al., 2019). The translational relevance to psychiatric conditions, mostly anxiety disorders, has been the main driving force behind a wealth of animal research literature on a variety of experimental conditioning designs, including TAA, and their neurobiological underpinnings reverberating around the amygdala, the key orchestrator of CS–US coupling, and of the ensuing CS-induced defensive freezing reactions (Maren, 2005; Johansen et al., 2011; Izquierdo et al., 2016). Relatively less studied, but as clinically relevant, are the potential deficits in fear conditioning and avoidance in common neurological conditions such as epilepsy (Kemppainen et al., 2006; Medlej et al., 2019a,b).

Pavlovian contextual conditioning is a well-established learning conditioning paradigm (Phillips and LeDoux, 1992; Maren et al., 1997; Musumeci et al., 2009; Izquierdo et al., 2016) that is particularly disrupted in models of limbic seizures and behavioral deficits (Medlej et al., 2019a; Kemppainen et al., 2006). While a variety of discrete cues such as tone and light have been used to signal shocks in the form of a CS in TAA paradigms, contextual cues have not been employed. Even though shocks are not signaled by a tone in the Sidman TAA, the context in which shocks are delivered in that test does not change with shuttling as both chambers are contextually similar (Lazaro-Munoz et al., 2010). The context in a Sidman TAA does not, therefore, clearly function as a CS or a warning signal that is terminated by the avoidance behavior as would occur with a classical CS such as a tone in a standard TAA. Indeed, in a Sidman TAA, avoidance is likely predominantly driven by a scheduled shock program rather than by a contextual CS as it has been argued about various Sidman paradigms and the likely temporal discrimination processes at play in such tests (Sidman, 1962; Anger, 1963; Bolles and Riley, 1973). Incorporating in the TAA a specific context such as a visually modified chamber, the exposure to which is terminated with the avoidance behavior, would be helpful to better assess active avoidance to a contextual CS. Such an incorporation would broaden the behavioral learning outcomes of the TAA in the study of anxiety and PTSD, and would enhance its applications in animal models of neurological diseases commonly accompanied by anxiety and prominent contextual learning deficits, namely, the epilepsies and their cognitive and emotional neurodevelopmental comorbidities (Kemppainen et al., 2006; Medlej et al., 2019a,b). However, unlike the ease of performing the repetitive trials of tone or light stimuli exposure required for successful TAA testing, repetitive exposure to visual contextual cues in one of the compartments of the standard two-compartment shuttle box is challenging. Indeed, the animal may not spontaneously re-enter the contextually modified compartment of interest with a reasonable frequency, and within a reasonable test duration in order to reliably assess the shock-avoidance rate with an adequate number of context exposure trials. This technical challenge has likely limited the study of contextual conditioning to simpler paradigms like classical Pavlovian conditioning, one-way active avoidance, and passive avoidance (Fabbri et al., 2016). Here, we describe a modified TAA (MAA) test that we designed to overcome this challenge by incorporating contextual Pavlovian conditioning in a standard two-compartment rat shuttle box. In the left compartment, electrical foot shocks were signaled by a tone, whereas in the right compartment, foot shocks were cued by specific patterns and objects on the walls. We maintained a standard tone-signaled TAA testing paradigm in the left compartment of the shuttle box in order to preserve frequent shuttling between the two compartments and, therefore, to assure an adequate number of contextual CS exposure trials in the right compartment. In this novel MAA test, we aimed at combining the assessment of active context-cued shock avoidance in the right compartment and tone-signaled shock avoidance in the left compartment.

## Materials and Equipment

### Animals

Animal care and behavioral studies were approved by the Institutional Animal Care and Use Committee (IACUC) at the American University of Beirut (AUB). The IACUC at AUB operates in compliance with the public health service policy on the humane care and use of laboratory animals, and implements the Guidelines for the Care and Use of Laboratory Animals of the Institute for Laboratory Animal Research of the National Academy of Sciences. The illustrative results below refer to MAA testing initiated at postnatal day 40 on 15 Sprague–Dawley male rats. The number of animals was chosen based on prior literature on TAA testing (Moscarello and LeDoux, 2013). Animals were obtained from the animal care facility at AUB and housed in a room maintained on a 12-h light–dark cycle. Rats were placed in high-temperature polysulfone rectangular cages (L 42.5 cm, W 27.6 cm, and H 15.3 cm) (TechniPlast, West Chester, PA, United States), two rats per cage, and fed *ad libitum* (Teklad Diet, Envigo, Indianapolis, IN, United States). Both the housing and behavioral testing units are temperature-controlled rooms (21°C). Testing always started at 9 a.m.

### The Apparatus: A Modified Shuttle Box

**FIGURE 1:**
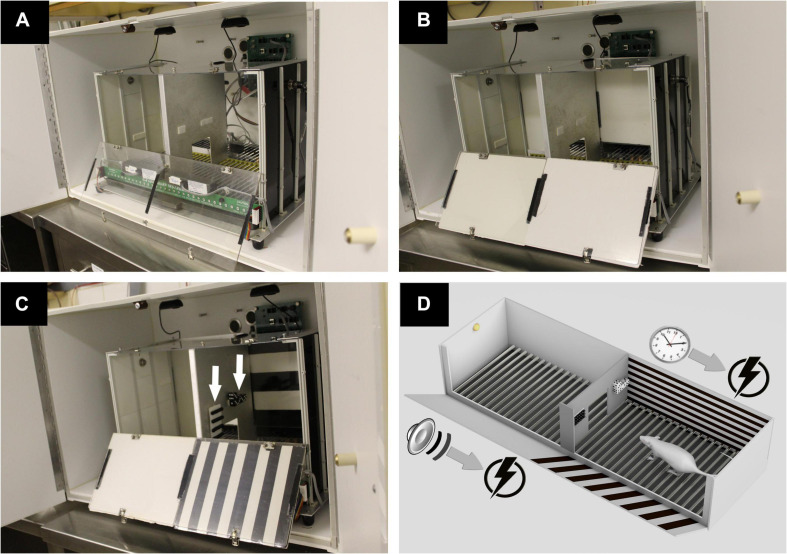
The apparatus and experimental protocol. **(A)** Shown is a shuttle box placed in a soundproof cubicle. The box consists of two chambers communicating through a door in the middle of a metallic partition wall. Electrical foot shocks are delivered via a stainless-steel grid floor. A transparent plexiglass plate allows video recording of the animal via two cameras centered above the right and left chambers. **(B)** During habituation on the first testing day, the anterior and posterior walls of both chambers are covered with white foam panels. **(C)** During training days, the left chamber remains unchanged from habituation day, whereas the right chamber is contextually modified with black and white striped foam panels and with four rows of beads and four dices (arrows) fixed on the right side of the partition wall. **(D)** A schematic representation of the experimental design during training days. In the left chamber, a trial consists of a 15-s tone that signals an incoming electrical foot shock. In the right chamber, an electrical shock is delivered for a maximum of 15 s following each 10 s spent in that compartment (permission obtained from *Epilepsy and Behavior, Elsevier*).

We used a standard two-way shuttle box (model H10-11R-SC, Coulbourn Instruments, Allentown, PA, United States) placed inside a soundproof isolation cubicle (H 51 cm, W 53 cm, and L 80 cm, model H10-24, Coulbourn Instruments, Allentown, PA, United States). The shuttle box consists of two identical chambers (H 34 cm, W 27 cm, and L 27 cm) that communicate through a 9 × 9-cm door in the middle of a metallic partition wall. The two-compartment box is equipped with a tone generator (4 kHz, 86 dB) and infrared beam sensors to detect transitions between its two compartments. Illumination is provided by two small light bulbs on each of the box sidewalls (model H11-05-LED, Coulbourn Instruments, Allentown, PA, United States). A precision animal shocker (model H13-15, Coulbourn Instruments, Allentown, PA, United States) delivers scrambled electrical foot shocks via a grid floor consisting of 32 parallel stainless steel bars. In order to video record the experimental rat through the ceiling and measure freezing, the shuttle box’s original metallic ceiling was replaced by a transparent plexiglass plate. Two high-definition USB cameras (model C310 HD, 720p/30fps, Logitech, Newark, CA, United States) were centered above the right and left chambers inside the isolation cubicle (Figure 1A), and the acquired videos were combined into a single one via a multiplexer software (3dtv.at, Germany). Modifications were also introduced in the right chamber by adding visual contextual cues on specific test days as described in the experimental protocol below. The cues consisted of two objects made of dices and beads attached to the right side of the metallic partition wall, as well as horizontally or vertically striped black and white foam panels placed on the anterior and posterior plexiglass walls of the right chamber. The sidewalls of the shuttle box consisted of white metallic plates (manufacturer-provided accessories).

## Methods

### The Experimental Protocol: Modified Active Avoidance

The MAA experimental protocol was programmed using the Graphic State 4.0 (GS4) software (Coulbourn Instruments, Allentown, PA, United States), which monitors the transitions between the left and right chambers and accordingly delivers tone signals and electrical foot shocks via the modular Habistest. The modular Habitest Linc and its USB adapter (models H02-08 and U90-11, Coulbourn Instruments, Allentown, PA, United States) are the factory-provided interface that connects the computer operating the GS4 experimental program to the shuttle box’s control board (model H03-04, Coulbourn Instruments, Allentown, PA, United States). The 7-day MAA test protocol consists of one habituation day, followed by 5 training days, and one retention test day as described below.

#### Habituation (Day 1)

The anterior and posterior plexiglass walls of each compartment are covered with plain white foam panels (Figure 1B). Rats are allowed to freely roam in the shuttle box for a duration of 5 min.

#### Training Days (Days 2–6)

**FIGURE 2:**
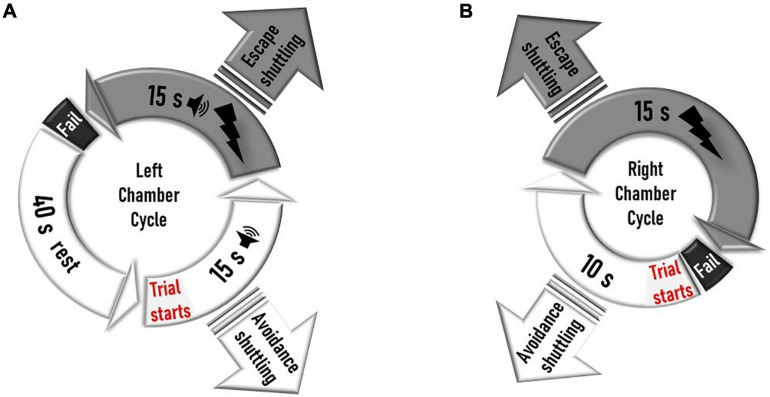
Schematic design of the left and right shock cycles during training days. **(A)** In the left chamber, a trial starts 40 s after the rat enters the chamber or 40 s after a failed trial on that side. The trial starts with a 15-s tone delivery, which is followed by a 15-s shock coupled to a tone. Shuttling to the right chamber during the 15-s shock-free tone delivery prevents the incoming shock (avoidance shuttling). Shuttling during the 15-s shock-delivery period aborts the ongoing shock (escape shuttling). If the rat does not shuttle during the shock, it fails the trial, and a new cycle starts after a 40-s intertrial rest period. If a rat shuttles to the right during the intertrial rest interval before a trial (tone delivery) is initiated in the left chamber, the rest period ends, and the right cycle is immediately initiated with a right chamber trial. **(B)** In the right chamber, the trial starts when a rat enters the chamber or immediately after a rat fails a trial on that side. Ten seconds after a trial starts, a 15-s shock is delivered. Shuttling to the left chamber during the 10-s shock-free interval prevents shock delivery (avoidance shuttling). Shuttling during the 15-s shock-delivery period aborts the ongoing shock (escape shuttling). If the rat does not shuttle during the shock, it fails the trial, and a new cycle starts. The training session on any given day finishes only when a total of 30 trials (left tone delivery or right chamber entry) are delivered.

Black and white striped foam panels are placed on the anterior and posterior plexiglass walls of the right chamber, while the left chamber remains unchanged from habituation day. Additional right chamber contextual cues made of dices and beads are attached on the right side of the interchamber partition wall (Figure 1C). In the contextually modified right chamber, an electrical foot shock (0.5 mA, 15 s) is delivered 10 s after entry to this compartment. Every entry to the right chamber is a context exposure trial. However, in the plain left chamber, a trial consists of a 15-s tone (CS) signaling an immediately succeeding 15 s of a 0.5-mA foot shock (US), with a 40-s intertrial period. Shuttling between chambers prevents an incoming shock (“avoidance”) or terminates an ongoing one (“escape”) (Figure 1D). Each of the five daily training sessions is concluded when a total of 30 trials are delivered (Figure 2).

#### Retention Test (Day 7)

After 5 days of training on associating the US (electrical foot shock) with an auditory CS in the left plain chamber, or with the conditioned right chamber contextual cues, a two-part retention test is performed. In the first part, the rat is allowed to freely roam for 2 min in the shuttle box without tone or foot shock delivery, in order to assess for the retention of context learning (the walls of the chambers and the visual cues are unchanged from training days). In the second part of the retention test, visual cues are removed from the right chamber, and the front and back walls are covered with white foam panels so that both the left and right chambers are identical and plain. Following 5 min of habituation, 30 trials of foot shocks (0.5 mA, 15 s), signaled by a preceding 15-s tone, are delivered in either the right or left chamber, with a 30-s intertrial period. Shock “avoidance” here again refers to preventing an incoming foot shock via shuttling through the interchamber door during tone delivery, and “escape” refers to aborting an ongoing foot shock via shuttling.

### Outcome Measures and Statistical Analyses

The rates of avoidance of, or escape from, shock delivery for the daily trials of left tone exposure or right context exposure are obtained from the experimental data recorded by the GS4 software. The latencies to avoid or to escape are also quantified using GS4. Moreover, freezing throughout the 5 days of training is assessed by analyzing the recorded video with the SMART video tracking 3.0 software (Panlab, Harvard Apparatus, Holliston, MA, United States). Freezing is defined as immobility with the absence of any movements except for those needed for breathing. The SMART software records the immobility time below a digitally set activity level threshold of 7 cm/s, and immobility epochs were counted only if immobility was present continuously for 5 s or more. These parameters were chosen as the detected freezing rates were consistent with those manually determined from the recorded videos in preliminary trials. The SMART software can also track the location of the rat in the left and right chambers, and, therefore, allows measuring freezing in either location independently. Statistical analyses were performed using Prism 7 (GraphPad Software, San Diego, CA, United States). Results were reported as means ± standard error of the mean (S.E.M.). One-way repeated measures analysis of variance (ANOVA) was used for the daily acquisition data, and the unpaired *t*-test for contextual retention, with a significance set at *p* < 0.05 for all comparisons.

## Illustrative Results

**FIGURE 3:**
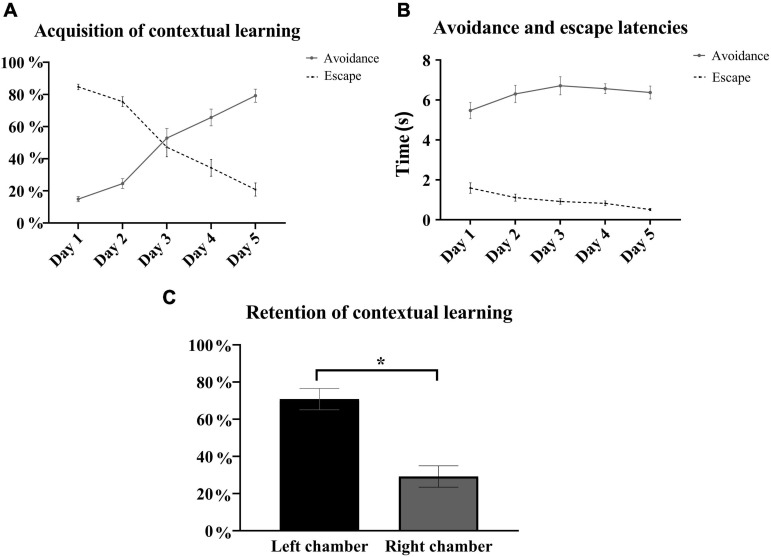
Contextual learning in the right chamber. **(A)** Presented is the learning curve of electrical foot shock avoidance and escape in the right contextually modified chamber over the 5 training days. Incremental increases in the average percentage of context-cued electrical foot shock avoidance were accompanied by gradual decrements in average escape rates with statistical significance in all comparisons between consecutive paired days in avoidance rates [*F*_(__3_, _41__)_ = 58.86, *p* < 0.05], as well as in escape rates [*F*_(__3_, _41__)_ = 57.17, *p* < 0.05] [repeated measures one-way-ANOVA with *post hoc* Fisher’s least significant difference (LSD), *n* = 15]. **(B)** Latencies to avoid and escape did not substantially change over the training days and ranged between 5.5 ± 0.4 and 6.7 ± 0.4 s, and between 0.5 ± 0.1 and 1.6 ± 0.3 s, respectively. **(C)** In the context retention subtest on day 6, all rats spent significantly more time in the left compartment (70.8 ± 5.7%) when allowed to freely roam [*t*_(__28__)_ = 5.12, *p* < 0.05, *n* = 15]. Mean ± SEM are reported.

In the contextually modified right chamber, rats shuttled during the electrical foot shock (escape) in more than 80% of the trials on the first training day, but this escape behavior was gradually replaced over the training days by shuttling prior to shock delivery or shock avoidance. During the 5 training days, there were daily statistically significant incremental increases in the percentage of context-cued electrical foot shock avoidance [one-way repeated measures ANOVA, *F*_(__3_, _41__)_ = 58.86, *p* < 0.05, *n* = 15] with a fivefold increase in the average avoidance rate from the first day (14.9 ± 1.6%) to the last training day (79.2 ± 4.1%). In parallel to the daily increases in the percentage of avoidance, there were daily decrements in the average escape rate from 84.7 ± 1.7% on day 1 down to 20.8 ± 4.1% on the last training day (Figure 3A). Rats successfully shuttled prior to (avoided) or during (escaped) shock delivery, with very low, less than 1% rates of failed trials (no avoidance or escape) on day 1 and none over the subsequent training days. While avoidance occurred within 5–7 s of exposure to the contextual stimuli, escape latencies were briefer, occurring within 2 s of shock delivery (Figure 3B). Retention of contextual learning was reflected in a statistically significant preference for the left compartment during retention testing [*t*_(__28__)_ = 5.12, *p* < 0.05, *n* = 15] (Figure 3C).

**FIGURE 4:**
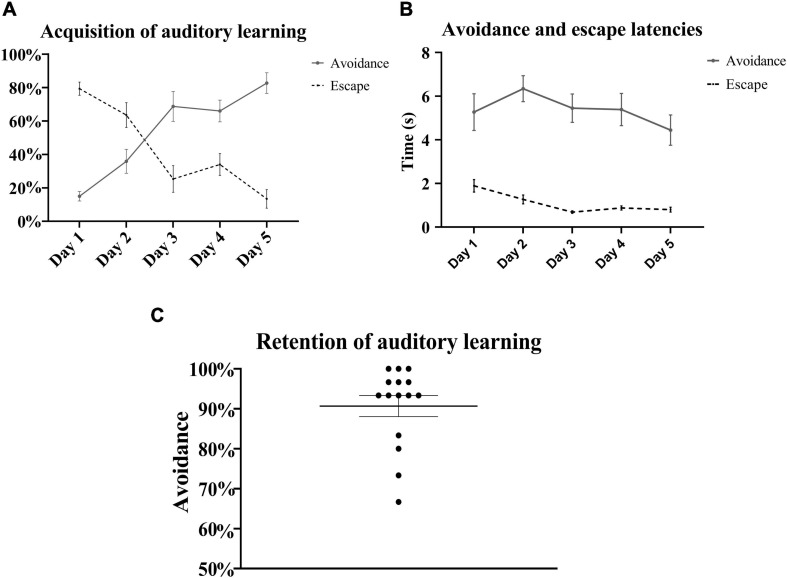
Auditory learning in the left chamber. **(A)** The learning curve of tone-signaled electrical foot shock avoidance and escape in the left compartment over the 5 training days. There were daily statistically significant incremental increases in the average percentage of tone-signaled electrical foot shock avoidance in all consecutive paired-day comparisons [repeated measures one-way ANOVA with *post hoc* Fisher’s least significant difference (LSD), *F*_(__3_, _48__)_ = 25.63, *p* < 0.05, *n* = 15] except between days 3 and 4 (*p* = 0.75). Those increases in avoidance rates were accompanied by gradual decrements in the average percentage of escape with statistical significance between days 2 and 3, as well as days 4 and 5 [repeated measures one-way ANOVA with *post hoc* Fisher’s LSD, *F*_(__4_, _51__)_ = 24.58, *p* < 0.05, *n* = 15]. **(B)** Latencies to avoid and escape did not substantially change over the training days, and ranged between 4.4 ± 0.7 and 6.3 ± 0.6 s, and between 0.7 ± 0.1 and 1.9 ± 0.3 s, respectively. **(C)** In the auditory learning retention subtest on day 6, all rats reached a high average tone-signaled shock avoidance rate of 90.7 ± 2.6%. Mean ± SEM are reported.

**FIGURE 5:**
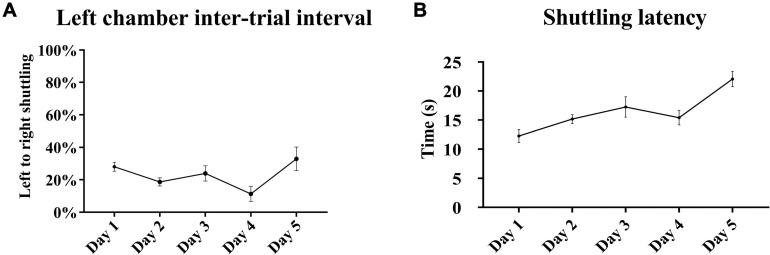
Shuttling during the left chamber intertrial interval. **(A)** The rates of left to right shuttling during the 40-s intertrial rest interval in the left chamber did not increase over the 5 training days. Minor fluctuations were observed with a drop reaching statistical significance on day 4 compared with days 1 and 5 [repeated measures one-way ANOVA with *post hoc* Fisher’s least significant difference (LSD), *F*_(__4_, _70__)_ = 3.15, *p* < 0.05, *n* = 15]. **(B)** There were no substantial changes in shuttling latencies, but there was a minor tendency for an increase over the training days reaching statistical significance between day 5 and all other days [repeated measures one-way ANOVA with *post hoc* Fisher’s LSD, *F*_(__4_, _70__)_ = 8.02, *p* < 0.05, *n* = 15]. Mean ± SEM are reported.

A pattern similar to the gradual acquisition of context-cued avoidance was observed with the tone-signaled trials in the left chamber. There were daily statistically significant incremental increases in the avoidance of tone-signaled electrical foot shocks from 15 ± 2.9% on the first training day up to 82.7 ± 6.2% on the last day [one-way repeated measures ANOVA, *F*_(__3_, _48__)_ = 25.63, *p* < 0.05, *n* = 15], with a concomitant gradual decrease in the rate of escape from 79.3 ± 3.9% on the first training day down to 13.4 ± 5.5% on the last day (Figure 4A). The rates of failed trials in avoidance or escape did not substantially change over the training days and ranged between 0 and 6%. Avoidance occurred within 4–7 s of tone initiation, and escape occurred within 2 s of shock initiation, with no substantial changes in these latencies over the 5 training days (Figure 4B). Tone-signaled shock avoidance latencies were statistically comparable with the latencies to avoid the context-cued shocks in the right chamber on days 1–4, but were lower by a minimal 1.9 s on day 5 [two-way repeated measures ANOVA, *F*_(__28_, _112__)_ = 2.41, *p* < 0.05, *n* = 15]. In the auditory retention subtest, retention of learning was reflected in a higher than 90% rate of tone-signaled shock avoidance in more than 70% of rats (average avoidance of 90.7 ± 2.6%) when auditory CS was tested in both chambers on the last test day, in the absence of contextual cues in a similar fashion to a standard TAA test (Figure 4C). Analysis of shuttling rates and latencies during the intertrial rest interval in the left chamber revealed no substantial changes over the 5 training days (Figure 5).

**FIGURE 6:**
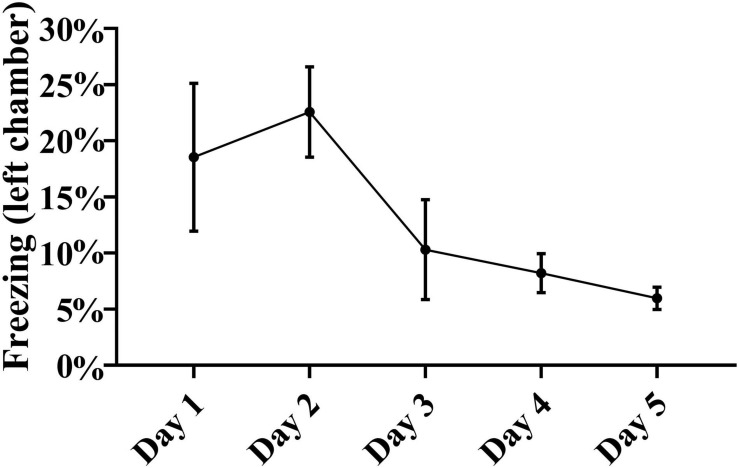
Freezing in the left chamber. Presented is the duration of freezing behaviors over the 5 training days in the left chamber, where electrical foot shocks were signaled by a tone. Because freezing requires hours of video analyses per rat, it was only performed on a relatively small illustrative sample of five rats randomly chosen from the initial cohort of 15 animals. The duration of freezing gradually decreased over the 5 training days. Repeated measures one-way ANOVA with *post hoc* Fisher’s least significant difference (LSD) did not reveal significance in this small illustrative sample of five animals, but there was significance in paired ANOVA comparisons when freezing duration on the second day of testing was compared with freezing duration on the fourth day, or with the fifth day [*F*_(__2_, _9__)_ = 3.05, *p* < 0.05, *n* = 5].

In order to further analyze the acquisition of learning, we also assessed whether there were any CS-induced defensive freezing reactions during the training days, by measuring immobility level, a surrogate of freezing. Analysis of immobility revealed the presence of defensive freezing reactions in around 20% of the time spent in the left chamber in the first 2 training days, followed by a gradual drop to less than 10% in the last 2 days (Figure 6). In the right chamber, freezing analysis revealed only a minimal less than 2% freezing on the first training day (1.8 ± 0.8%), with a drop to less than 1% on days 2–4, and no detectable freezing on the last training day.

## Discussion

Here, we describe a novel MAA test that combines testing of active tone-signaled electrical foot shock avoidance and active context-cued shock avoidance in a two-way shuttle box. Easy-to-implement yet key modifications to the standard shuttle box allowed combining these two testing paradigms in our MAA test, without requiring a higher number of animals or experimental trials than a standard TAA test. Our novel MAA test, therefore, efficiently expands the TAA array of outcomes and broadens its applications in mechanistic studies and translational research.

The herein described novel procedure efficiently combines contextual and auditory active avoidance by providing repetitive exposure to a contextual CS that consists of a visually modified chamber with object and stripe patterns on its walls. Combining this contextual CS in one of the chambers of a shuttle box with an auditory CS in the other chamber maintained the mobility of the rat between the two chambers. Indeed, escape from, or avoidance of, the repetitive programmed delivery of tone-signaled shocks in the plain chamber assured the return of the rat to the contextually modified chamber, and thus, a repetitive exposure to the contextual CS. This combination overcame the technical challenge of repeatedly exposing an animal to contextual cues in an automated manner, and thus, provided a sufficient number of contextual exposure trials for meaningful statistical analyses. Successful learning acquisition was evidenced by the gradual statistically significant incremental increases in shock avoidance rates in each chamber over the 5 training days. As opposed to only a short 10-s intershock interval in the contextually modified chamber, a relatively prolonged rest period of 40 s followed by 15 s of tone delivery preceded foot shocks in the plain chamber, successfully resulting in the pairing of US–CS in the form of a context–shock association in the visually modified chamber and a tone–shock association in the plain one, with comparable learning curves and daily increments in avoidance rates in both chambers. Furthermore, a nearly constant 5- to 6-s latency to avoid was observed in all the trials in which rats avoided shocks, in either chambers, and on all training days (Figures 3B, 4B), suggesting that avoidance shuttling actively occurs at “a right time” following threat detection, and is not a passive or random behavior. While a shock schedule may unconditionally drive avoidance behaviors in a schedule-dependent manner in the absence of a discrete warning signal (Sidman, 1953, 1962; Anger, 1963; Bolles and Riley, 1973), it is unlikely that the shock schedule in our MAA resulted in scheduled behaviors since a similar 5- to 6-s latency to avoid occurred after exposure to tone in the plain chamber or to context in the visually modified chamber, despite the differences in shock cycle schedules between the two chambers. The contextual cues and the tone were, therefore, the warning signals that governed avoidance behaviors and the “right time to avoid.” Indeed, the contextual visual cues and the tone became independent threat scenarios or CS as evidenced by the avoidance of the visually modified chamber and the high rates of avoidance of tone-signaled shocks in a plain box when the two CS were separately tested in the last-day retention subtests. The freezing reactions observed in the early training days also support the fact that both context and tone became independent CS. Our setup and box modifications allowed the automated digital measurement of immobility, a surrogate of freezing reactions, independently in the right and left chambers. Only minimal freezing was detected in the contextually modified chamber and exclusively during the early training days, likely due to the lack of epochs of immobility that are prolonged enough to be detected and the relatively rapid replacement of freezing by adaptive avoidance. Indeed, a gradual drop in freezing in both chambers paralleled the increase in shock avoidance rates. This replacement of *reactive innate* defensive freezing reactions by *active learned* adaptive avoidance behaviors is in line with the previously reported elegant experiments in the standard TAA test (Moscarello and LeDoux, 2013; Campese et al., 2014).

Compared with the standard TAA test, the herein-employed MAA provides a richer array of outcomes without subjecting rats to additional painful electric foot shocks, and with a comparable number of animals, and number of trials and test days, to currently used TAA protocols (Moscarello and LeDoux, 2013). Moreover, the substantial learning rates in both chambers of our MAA test were comparable with those described in the standard TAA and were higher than the learning rates in the Sidman TAA, which results in a substantial number of poor performers likely due to the lack of a warning signal in a Sidman paradigm (Lazaro-Munoz et al., 2010). Unlike the Sidman TAA, the presence of a warning signal in both chambers of our MAA promoted learning in both compartments. Indeed, entry to the contextually modified chamber signaled a severe shock schedule, and the exposure to this visually modified chamber was terminated by the avoidance behavior of shuttling to the plain chamber. This pairing rendered exposure to the contextually modified chamber a warning signal that drives avoidance learning in a similar manner to tone signaling in the plain chamber. Rats, however, did not develop avoidance behaviors to the context in which the tone was delivered (the plain chamber) over the 5 training days as evidenced by the lack of an increase in shuttling rates during the intertrial rest interval prior to tone delivery in the plain chamber. One limitation of our MAA paradigm is that contextual avoidance can only be accurately studied during the training phase of the test. Indeed, while the contextual retention subtest supports the fact that the contextually modified chamber becomes a CS during active avoidance training, the retention is scored based on the passive avoidance of that chamber. Because shocks are delivered in both chambers during training, but to a lesser extent in the plain one, the time of staying outside the visually modified chamber during our MAA contextual retention is relatively shorter than what is observed in passive avoidance protocols (Kaminsky et al., 2001; Fabbri et al., 2016). Another potential limitation in our MAA paradigm is the codependence of the left and right testing, in that shuttling from one chamber is required to obtain an appropriate number of trials in the other chamber. However, even during the first test session, the untrained rats had high rates of successful right and left shuttling (above 99% and 94%, respectively) mostly in the form of an escape shuttle early during shock delivery. The left-right codependence is, therefore, unlikely to have a substantial effect on the use of our MAA. Lastly, a minor limitation in this work is not counterbalancing the side with the experimental condition.

Our novel MAA efficiently incorporates a contextual CS in a standard TAA test and assesses the progression of animal behavior from reactive freezing and escape to learned avoidance of a contextual CS in the visually modified chamber or an auditory CS in the plain one. Incorporating contextual conditioning in a shuttle box renders the TAA richer in its array of behavioral outcomes and makes it a useful complementary test to facilitate comparisons between two learning modalities in the same experimental animal, in order to potentially better dissect the neural circuitry regulating avoidance, specifically the contribution of the hippocampus, given its well-established role in contextual learning (Phillips and LeDoux, 1992; Maren et al., 1997; Musumeci et al., 2009; Izquierdo et al., 2016). Indeed, despite the elucidation of many aspects of the neuronal circuitry governing TAA behaviors (Maren, 2005; Moscarello and LeDoux, 2013; Izquierdo et al., 2016; LeDoux et al., 2017; Moscarello and Maren, 2018), the exact role of the hippocampal circuitry in this test awaits more research (Moscarello and Maren, 2018). Along those lines, ongoing work in our laboratory is aiming at assessing the effects of lesional and/or functional hippocampal deficits on the different MAA outcomes. From a translational end, despite the difference in shock cycles between the right and left chambers of our MAA test, the balanced comparable auditory and contextual learning curves renders it a reliable testing platform to assess the effects of variable brain insults on the clinically relevant outcome of avoidance behaviors. While threat avoidance intuitively seems to be adaptive and subserving animal survival, it can be maladaptive and socially impairing when excessive in certain anxiety disorders such as phobias and PTSD. The MAA, along with other tests, can assist in devising strategies to potentially attenuate avoidance when maladaptive (Maren, 2005), or preserve its normal function when altered by amygdalo-hippocampal insults in human diseases such as epilepsy, and particularly during cognitive and emotional neurodevelopment as illustrated in our prior work (Medlej et al., 2019a,b). Indeed, clinically as well as in animal models, in addition to seizures, amygdalo-hippocampal epileptogenic insults often lead to anxiety and cognitive disturbances (Thome-Souza et al., 2004; Obeid et al., 2010; Verrotti et al., 2014; Medlej et al., 2019a) with predominant deficits in contextual fear conditioning in rodent models (Kemppainen et al., 2006).

In conclusion, our novel MAA test efficiently combines contextual and auditory active avoidance testing in a shuttle box without requiring a higher number of animals or experimental trials than a standard TAA test. Efficiently combining these two testing paradigms in our MAA test was allowed by simple-to-implement yet key modifications that provided repetitive trials of context exposure in one of the chambers of a shuttle box, while maintaining a standard TAA testing paradigm in the other chamber. In this work, the MAA was conducted on periadolescent rats in order to establish its use at the neurodevelopmental stage that is predominantly investigated in our laboratory. Ongoing studies by our team aim at assessing the use of this test at various age groups and the effects of hippocampal pathology on its different behavioral outcomes in rat seizure models. By incorporating contextual conditioning in the two-way shuttle box, and efficiently expanding its array of outcomes, our novel MAA test will be a useful complement to the standard TAA in mechanistic studies, and will enhance its application to rodent models of neurological conditions accompanied by anxiety and learning deficits.

## Data Availability Statement

The data supporting the conclusions of this article will be made available by the authors, without undue reservation.

## Ethics Statement

The study was reviewed and approved by the Institutional Animal Care and Use Committee (IACUC) at the American University of Beirut.

## Author Contributions

HS, YM, and RA performed the experiments. HS, MD, and YM analyzed the data. HS, RAR, YM, and RA wrote the original draft. YM and HH curated the data. HH wrote the first draft. SD and YM performed the freezing analyses. CF, HH, and MO were in charge of the visualization. MO conceptualized the study, developed the methodology, wrote—reviewed and edited the article, and was in charge of supervision, project administration, and funding acquisition. All authors contributed to the article and approved the submitted version.

## Conflict of Interest

The authors declare that the research was conducted in the absence of any commercial or financial relationships that could be construed as a potential conflict of interest.
